# Faster Movement Speed Results in Greater Tendon Strain during the Loaded Squat Exercise

**DOI:** 10.3389/fphys.2016.00366

**Published:** 2016-08-31

**Authors:** Jacob E. Earp, Robert U. Newton, Prue Cormie, Anthony J. Blazevich

**Affiliations:** ^1^Human Performance Laboratory, Department of Kinesiology, University of Rhode IslandKingston, RI, USA; ^2^Centre for Exercise and Sports Science Research, School of Medical and Health Sciences, Edith Cowan UniversityJoondalup, WA, Australia; ^3^Institute for Health and Ageing, Australian Catholic UniversityMelbourne, VIC, Australia

**Keywords:** viscoelastic, quadriceps, patellar, tendon, rate of force development, fascicle, Young's modulus

## Abstract

**Introduction:** Tendon dynamics influence movement performance and provide the stimulus for long-term tendon adaptation. As tendon strain increases with load magnitude and decreases with loading rate, changes in movement speed during exercise should influence tendon strain.

**Methods:** Ten resistance-trained men [squat one repetition maximum (1RM) to body mass ratio: 1.65 ± 0.12] performed parallel-depth back squat lifts with 60% of 1RM load at three different speeds: slow fixed-tempo (TS: 2-s eccentric, 1-s pause, 2-s concentric), volitional-speed without a pause (VS) and maximum-speed jump (JS). In each condition joint kinetics, quadriceps tendon length (L_T_), patellar tendon force (F_T_), and rate of force development (RFD_T_) were estimated using integrated ultrasonography, motion-capture, and force platform recordings.

**Results:** Peak L_T_, F_T_, and RFD_T_ were greater in JS than TS (*p* < 0.05), however no differences were observed between VS and TS. Thus, moving at faster speeds resulted in both greater tendon stress and strain despite an increased RFD_T_, as would be predicted of an elastic, but not a viscous, structure. Temporal comparisons showed that L_T_ was greater in TS than JS during the early eccentric phase (10–14% movement duration) where peak RFD_T_ occurred, demonstrating that the tendon's viscous properties predominated during initial eccentric loading. However, during the concentric phase (61–70 and 76–83% movement duration) differing F_T_ and similar RFD_T_ between conditions allowed for the tendon's elastic properties to predominate such that peak tendon strain was greater in JS than TS.

**Conclusions:** Based on our current understanding, there may be an additional mechanical stimulus for tendon adaptation when performing large range-of-motion isoinertial exercises at faster movement speeds.

## Introduction

Muscular forces are transmitted via tendons to the skeleton, and this transmission typically confers both movement economy and power benefits (Ettema, 1996, 2001; Bobbert, 2001). Tendons therefore modulate the movement output response for a given muscular input, with this influence being governed by the tendon's mechanical properties (Ettema, 1996; Bobbert, 2001; Lichtwark and Wilson, 2007). It is well-accepted that a tendon's properties adapt in response to chronic changes in loading volume and intensity (Kubo et al., 2001; Arampatzis et al., 2007; Kongsgaard et al., 2010). The possibility therefore exists to manipulate loading in order to elicit specific adaptive responses in the tendon and control (i.e., optimize) the muscle input-movement output relationship.

Tendon strain evoked by an imposed stress is believed to provide the primary mechanical stimulus for tendon adaptation and this can be easily imposed, and therefore manipulated, during resistance-type exercise (Arampatzis et al., 2007, 2010). Consequently, an understanding of the effects of changes in exercise loading intensity (including movement speed) and movement pattern (e.g., joint range of motion), particularly in commonly-performed but functionally complex multi-joint exercises, would provide the opportunity to control the mechanical stimulus and thus the tendon adaptive process. A significant issue, however, is that the prediction of tendon stress-strain patterns during complex movements is difficult. This is because tendons are viscoelastic structures that exhibit both rate dependent (viscous) and rate independent (elastic) properties so changes in loading magnitude and rate differentially affect the stress-strain relationship, and changes in movement strategy (e.g., movement speed or joint ranges of motion) alter the relative joint moment and power contributions to a task, and thus alter the stress imposed on different tendons (Arampatzis et al., 2007; Abellaneda et al., 2009).

With specific regard to the influence of a tendon's viscoelastic properties, a greater load during isometric contractions is seen to elicit an observable and predictable tendon strain pattern, consistent with the elastic properties of tendon (Arampatzis et al., 2007). However, an increased tendon strain velocity resulting from faster muscular force development has been shown to increase tendon stiffness, and thus reduce strain at a given level of force (Arampatzis et al., 2010). In fact, recent evidence suggests that tendinous tissue strain during explosive movements may be minimal when very high forces are produced as rapidly as volitionally possible (Earp et al., 2014). These findings are of great interest because they not only indicate that a tendon's elastic energy storage and release potential during a movement may be influenced by loading rate, but because they indicate that a tendon's loading rate might influence its chronic adaptation (Arampatzis et al., 2007, 2010). It may be speculated, therefore, that increases in strain magnitude (i.e., loading magnitude) might stimulate increases in tendon stiffness but that increases in the strain rate (i.e., loading rate) may reduce this adaptive response (Roeleveld et al., 1993; Kubo et al., 2001; Arampatzis et al., 2007, 2010; Bohm et al., 2014). However, to our knowledge no studies have directly compared tendon adaptation when external load is fixed and tendon strain is manipulated by varying movement velocity.

Several studies have examined the effect of movement speed on muscle and tendon dynamics during running and jumping exercises (Finni et al., 2001, 2003; Ishikawa et al., 2003, 2007). In these studies it was concluded that tendon strain increases and muscle strain decreases as movement speed, and thus the muscular (and tendon) forces, increases (Finni et al., 2001, 2003; Ishikawa et al., 2003). Such muscle-tendon unit behavior allows the tendon to act as an elastic catapult, during which movement economy and muscular power are increased (Gosset et al., 2009). These studies also indicate that the tendon's elastic properties predominately influence how the muscle and tendon interact, and that these properties ultimately affect tendinous tissue behavior and thus movement performance. In contrast, the viscous properties clearly had no detectable influence. Nonetheless, whilst running and jumping movements are common in sports and daily activities, they are relatively ineffective for stimulating tendon adaptation when compared to resistance exercise and are therefore not commonly used with the specific aim of altering tendon properties (Hansen et al., 2003; Arampatzis et al., 2007; Gosset et al., 2009; Lichtwark et al., 2013; Bohm et al., 2014). Exercises involving large muscular forces performed through a large range of motion at slower movement speeds, however, are commonly used to influence tendon stiffness or treat tendinopathy (Kongsgaard et al., 2010; Malliaras et al., 2013; Joseph and Denegar, 2015; Wiesinger et al., 2015). Because, the running and jumping exercises performed in those previous studies did not utilize additional loading strategies, and were performed through relatively small ranges of motion and for short (total time) durations, these data do not allow speculation as to how tendons might behave during loaded multi-joint movements such as squat lifts or loaded jumps (Hansen et al., 2003; Arampatzis et al., 2007; Gosset et al., 2009; Lichtwark et al., 2013; Bohm et al., 2014). Thus, it is unknown how speed of movement might influence both tendon stiffness and tendon health during exercises commonly prescribed to elicit tendon adaptation (Kongsgaard et al., 2010; Malliaras et al., 2013; Joseph and Denegar, 2015; Wiesinger et al., 2015).

Presently, it remains unknown as to how the manipulation of factors such as movement speed and range of motion during commonly used isoinertial resistance training exercises influences muscle-tendon strain patterns. Since peak tendon strain has been hypothesized to drive tendon adaptation (Arampatzis et al., 2010), and because movement velocity is an easily manipulated acute programming variable during resistance training, this information may enhance our understanding of tendon rehabilitation and prehabilitation programming. Alternatively, peak tendon load (i.e., the force expressed through the tendon) may be the predominate determinant of tendon adaptation (Arampatzis et al., 2007). Thus, research is necessary to differentiate movements that emphasize tendon strain over tendon load and vice versa so comparisons of chronic adaptation between such movements can be compared.

In the present study, we specifically examined how the manipulation of movement speed influences patellar tendon dynamics during squatting exercises. We hypothesized that tendon strain would be greatest when heavy loads were lifted in squats performed at a slow speed and fixed tempo (Tempo Squat: TS) compared to squats with maximal volitional speed (i.e., Jump Squat: JS), where the viscous properties of the tendon would minimize the resulting tendon strain. Furthermore, we hypothesized that the performance of heavy squats performed at a slow speed and fixed tempo (TS) would result in greater tendon strain than when the same exercise was performed at self-selected (volitional) speed (VS) without jumping.

## Materials and methods

### Subjects

Ten healthy, resistance-trained men, who habitually performed loaded squat and jump exercises (age: 25.8 ± 2.8 years, height: 1.77 ± 0.06 m, body mass: 83.8 ± 9.4 kg) participated in the study. Subjects were screened to ensure that their one-repetition maximum (1RM) for the parallel-depth squat exercise was at least 1.5 times body mass [(BM); 1RM = 138 ± 16 kg, 1RM:BM = 1.65 ± 0.12], that they did not have a recent history (2 years) of lower body musculoskeletal injury or tendinopathy, and that they were able to perform maximal effort lifts. The study was reviewed and approved by the Human Research Ethics Committee of the University and all subjects gave their informed written consent prior to participation.

### Experimental design

After being familiarized with the experimental protocol the subjects had their 1RM determined for the parallel-depth back squat exercise. On a separate day 3–7 days later the subjects completed the experimental session. Subjects were required to refrain from any exercise for at least 72 h and refrain from alcohol and caffeine for at least 24 h before both 1RM testing and the experimental session. During the experimental session, the subjects performed parallel-depth fixed-tempo squats (TS; described below), volitional speed squats (VS), and maximal velocity jump squats (JS) with an external load of 60% of their 1RM in a randomized order. At each speed, elongation of the distal vastus lateralis (VL) tendinous tissue and patellar tendon (i.e., tendon dynamics), movement kinetics and kinematics, and VL muscle activity were captured using integrated high-speed ultrasonography and motion analysis, a tri-axial force platform, and surface electromyography (sEMG). As the purpose of the present study was to quantify the effect of movement speed on tendon dynamics, comparisons were made between squat lift exercises performed with equal load (60% of back squat 1RM) and identical squat depth but with varying concentric speeds (TS < VS < JS).

### Movement technique

The subjects were required to perform all squat exercises to a parallel depth, which was defined as the femoral line being parallel to the ground. Prior to initial testing, the subjects performed a parallel-depth squat with a plastic bar, during which bar displacement was measured using a ceiling-mounted linear position transducer (PT5A-150, Celesco Transducer Products, Chatsworth, CA) attached 10 cm from the bar's center and right knee flexion angle was measured using an electrogoniometer (MLTS700, AD Instruments USA) sampling at 1000 Hz. Maximal knee flexion angle and minimum bar displacement achieved during this squat were then used as the standard depth for all subsequent squats and jump squats. Data were displayed in real time during testing and repetitions in which peak knee flexion deviated ± 2.5° or bar displacement deviated ± 1.25 cm from the standard were not accepted. Verbal and visual feedback was given to the subjects after each repetition to improve consistency.

### One-repetition maximum

The subjects completed a standardized warm-up prior to 1RM testing. This warm-up consisted of 5 min of low-intensity cycling (5 kP, 60 rpm) on a cycle ergometer (Erogomedic 839, Monark, Sweden) before four sets of submaximal squats were performed with a 2 min inter-set rest and with loads imposed according to their 1RM (10 repetitions at 0% 1RM, 8 repetitions at 50% 1RM, 3 repetitions at 80% 1RM, and 1 repetition at 90% 1RM). Subjects were given 3 min of rest between 1RM attempts and all subjects obtained their 1RM within six attempts (Baechle and Earle, 2008).

### Squat and jump squat testing

Prior to the experimental session the subjects performed the standardized warm-up described above with the addition of one set of three submaximal jump squats with no external load and with increasing jump height (one jump each at 30, 60, and 90% of perceived maximal intensity). This was followed by 1 min of rest and one maximal intensity jump squat with no external load. After completing the warm-up, the subjects performed TS, VS, and JS conditions with an external load = 60% of their 1RM in a randomized order. At the completion of testing an additional maximal unloaded jump squat was performed to determine if the protocol elicited a significant fatigue effect. *Post-hoc* comparison of vertical jump height, as measured during unloaded jump squats before and after testing, revealed no difference in performance (paired *t*-test, *p* > 0.05); therefore fatigue was not considered to have influenced the results. Possible order effects were checked by comparing the relative jump height and peak tendon strain during JS when performed as the first, second or third condition, and no effect was found (one-way ANOVA, *p* > 0.05).

During TS the subjects were required to squat at a constant, slow speed and hold the lowest position in the squat for 1-s (2-s eccentric phase, 1-s pause, 2-s concentric phase). Movement speed was controlled using a metronome and repetitions not matching these criteria were discarded and the repetition repeated after 1 min of passive rest. During VS the subjects squatted using a self-selected (i.e., volitional) movement speed but were instructed not to pause during the transition between the eccentric and concentric phases (i.e., the amortization phase). During JS the subjects were instructed to “Jump as high as possible while keeping the load in constant contact with your shoulders” but no other instruction was provided, thus velocity of the eccentric phase could be selected by the subjects.

For each exercise the subjects performed at least two repetitions, each separated by 1 min of passive rest, and 2 min of passive rest was imposed between conditions. Additional repetitions were performed if the knee angle or bar displacement did not match the testing criteria previously outlined. No subjects required more than two additional repetitions at any speed. For each exercise, the repetition that most closely matched the knee angle and bar displacement achieved during 1RM testing was selected for further analysis.

### Surface electromyogram (sEMG) collection and analysis

sEMG signals were recorded from VL (sEMG_VL_), vastus medialis, rectus femoris, biceps femoris, gluteus maximus, medial gastrocnemius, soleus, erectore spinae, and tibialis anterior as per SENIAM guidelines using self-adhesive surface electrodes (Meditrace, Tyco Healthcare, Australia) placed in a bipolar configuration over the muscle belly on the left side of the body (Winter, 1990; Hermie and Freriks, 1996). Inter-electrode distance was set at 2 cm and all signals were checked to ensure inter-electrode impedance was < 5 kΩ. Raw signals were recorded using a wireless sEMG system (ZeroWire, Aurion, Milan Italy) at an analog-to-digital conversion rate of 2000 Hz and 16-bit resolution after being amplified (1000x). Recorded signals were full-wave rectified and filtered using a dual-pass, 6th order, 10–500 Hz band-pass Butterworth filter, and then a linear envelope was created using a zero-lag, low-pass, 2nd order Butterworth filter with a cut-off frequency of 6 Hz. sEMG signals recorded during each repetition were normalized to the peak muscle activity recorded during a 0% 1RM (i.e., body weight) jump squat to volitional depth. This allowed signals to be reported as a percentage of the maximal activity observed during the 0% 1RM jump squat. This normalization procedure was implemented as pilot testing demonstrated that all muscles investigated were active with repeatable amplitudes during this task.

### Movement kinetics

The subjects performed all exercises in the experimental session whilst standing on a calibrated tri-axial force platform sampling at 1000 Hz (9290AD, Kistler Instruments, Winterhur Switzerland) with a synchronized high-speed camera (Sony HDV CRX 4100, USA) recording at 100 fps (2000 Hz shutter speed) placed 2.5 m lateral to the center of the bar, and used to record all movements. Reflective markers were placed on the subjects' left sides at the 5th metatarsal head, lateral border of the calcaneous, lateral malleolus of the fibula, distal lateral epicondyle and greater trochanter of the femur, and the side of the neck at the level of the 5th cervical vertebra. A 4-segment model was created using the X–Y coordinates of each marker. Positions of the ankle, knee, and hip joint centers were calculated throughout the movement as well as the minimum and maximum relative joint angles, and angular velocities and accelerations.

The relative ankle, knee, and hip joint moments were estimated by combining force platform and kinematic using standard inverse dynamics equations and with segmental masses estimated using the cadaver-derived equations provided by Dempster et al. (Dempster, 1955; Robertson et al., 2004). Frontal plane kinetics and kinematics were assumed to be less important and were therefore not assessed, and thus it was assumed that joint movements were identical between left and right sides (Robertson et al., 2004). Relative joint moments are reported after normalizing the absolute joint moments by the subjects' body masses.

Patellar tendon forces (F_T_) were estimated by multiplying knee moment by the joint-derived moment arm length of the patella; as determined using a previously published model (Visser et al., 1990). It should be noted that calculation of F_T_ using this model follows the assumption that joint torque from knee extensors other than the quadriceps femoris and from the knee flexors was negligible. Rate of force development in the tendon (RFD_T_; i.e., produced in the quadriceps) was calculated as the gradient of the F_T_-time relationship at a given instant in time, after smoothing with a zero-lag, 5 Hz low-pass, 3rd order Butterworth filter (Cormie et al., 2008).

### Muscle-tendon unit (MTU) behavior

**Figure 1:**
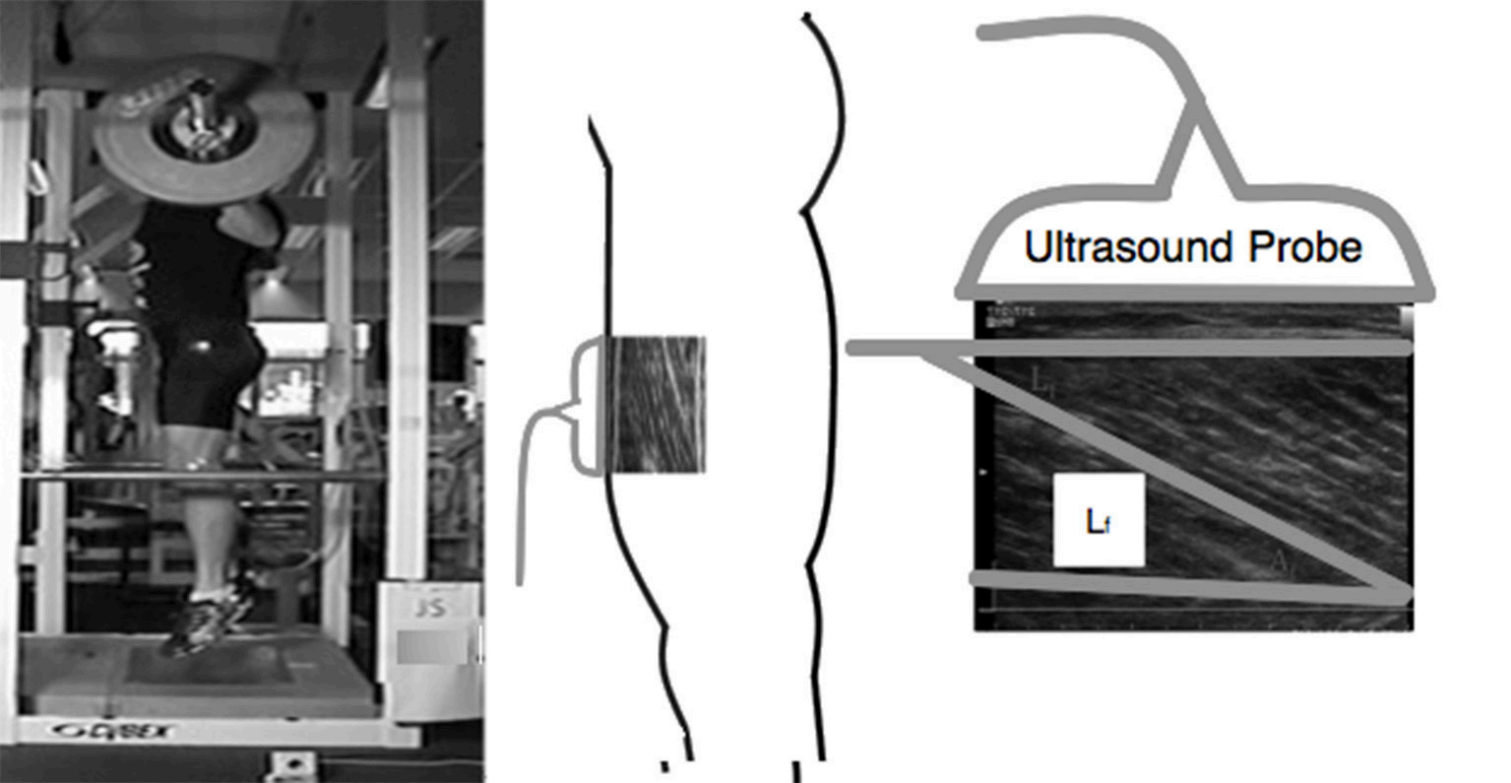
**Example of experimental design depicting how VL fascicle length was measured and extrapolated during a jump squat**.

MTU behavior was observed during the experimental session using high-speed (96 Hz) B-mode ultrasonography (Alpha 10, Aloka, Co Ltd, Tokyo, Japan). During TS, VS, and JS movements, longitudinal ultrasound images of the VL fascicles were collected using a 6 cm, 10 MHz, T-head linear array transducer (UST 5713, Aloka Co Ltd, Tokyo, Japan) which was placed at 50% of the distance between the greater trochanter and the lateral condyle of the femur (Figure 1). The transducer was aligned with the direction of the VL fascicles so that single fascicles could be tracked throughout the entire range of motion and fixed to the subject using a custom-made thermoplastic cast (Earp et al., 2014). A thin echo-absorbent reference strip was fixed to the subjects' skin under the transducer to allow for correction of any transducer movement that occurred during the testing.

The method by which fascicle length was estimated has been described in detail elsewhere (Earp et al., 2014) and has been found to be both valid and reliable (Kurokawa et al., 2001; Earp et al., 2014). Briefly, in each recorded image the visible fascicle length and fascicle angle were measured for three different fascicles and manually tracked throughout the movement using video analysis software (Prosuit 5.0, DartFish, Sydney, Australia). For each fascicle, non-observable portions were geometrically extrapolated and the calculated fascicle lengths of the three fascicles were then averaged. MTU length was estimated using previously derived models based on joint position and subject limb lengths (Ishikawa et al., 2003). The length of the tendinous tissue of the quadriceps tendon (L_T_) was calculated as the longitudinal length of the recorded fascicle subtracted from L_MTU_. L_T_ calculated with this method includes the distal and proximal tendon components (Kurokawa et al., 2001; Abellaneda et al., 2009).

### Data collection and processing

Bar displacement and sEMG data were recorded simultaneously using the PowerLab data acquisition system and associated software (PowerLab 16/35 and LabChart 7.2, AD Instruments, Bella Vista Australia). Ultrasound images were synchronized using a 5 V output pulse triggered by the data acquisition software and sent to the ultrasound system, which was also used to initiate force plate recording (Earp et al., 2014). High-speed video capture was synchronized with the data acquisition system using a custom-made one-way light switch.

All signal filtering and analyses were performed using custom-written LabView programs (Labview 8.2, NI Instruments, Texas, USA). To allow for comparison between movements with different durations, signals were time-normalized and represented as a percentage of the movement between the start of the eccentric phase of the movement to the completion of the concentric phase (or when the foot left the force plate during the JS condition: 30).

### Statistical analysis

Repeated measures MANOVAs with Bonferroni *post-hoc* tests were used to examine differences in tendon dynamics (L_T_, F_T_, and RFD_T_), movement kinetics and kinematics, and VL muscle activity between the TS, VS, and JS conditions. An additional repeated measures MANOVA was used to examine differences in L_T_, F_T_, and RFD_T_ through the duration of the movement using the time-normalized tendon length curves. Results are presented as mean ± standard deviation. All statistical analyses were conducted using PASW 18.0.1 (IBM, New York, USA) and statistical significance was accepted at an α-level of *p* < 0.05.

## Results

Joint kinematic and kinetic data are reported in Table 1. Between-condition comparisons revealed no significant differences in joint range of motion or peak flexion of the ankle, knee, or hip joints, suggesting that a similar movement pattern was adopted in all conditions. However, movement velocity varied between conditions, with the angular velocity of the ankle, knee, and hip as well as bar velocity increasing in the order TS < VS < JS (*p* < 0.05) in both the eccentric and concentric phases, with the exception of the ankle where eccentric angular velocity was not significantly different between TS and VS (*p* = 0.215).

**Table 1:** **Joint kinetics and kinematics during slow-tempo (TS), volitional speed (VS), and jump (JS) squats performed with 60% of 1RM (***n*** = 10)**.

	**TS**	**VS**	**JS**
θ_a_(°)	86.6 ± 5.1	86.5 ± 6.2	84.2 ± 6.7
θ_k_(°)	77.6 ± 19.1	79.5 ± 19.4	76.6 ± 17.3
θ_h_(°)	82.0 ± 14.5	79.7 ± 15.4	78.2 ± 16.5
ω_a_ − Ecc (°s^−1^)	−44.7 ± 13.1^b^^c^	−58.8 ± 18.4^a^	−90.1 ± 54.7^a^
ω_a_ − Con (°s^−1^)	47.3 ± 9.3^b^^c^	73.1 ± 21.5^a^^c^	356.4 ± 89.5^a^^b^
ω_k_ − Ecc (°s^−1^)	−44.7 ± 13.1^b^^c^	−58.8 ± 18.4^a^^c^	−90.1 ± 54.7^a^^b^
ω_k_ − Con (°s^−1^)	128.1 ± 27.1^b^^c^	201.7 ± 40.4^a^^c^	449.6 ± 49.2^a^^b^
ω_h_ − Ecc (°s^−1^)	−109.6 ± 21.7^b^^c^	−149.4 ± 27.6^a^^c^	−165.6 ± 28.1^a^^b^
ω_h_ − Con (°s^−1^)	115.1 ± 28.6^b^^c^	180.1 ± 34.8^a^^c^	309.3 ± 54.7^a^^b^
T_a_ (Nm.kg^−1^)	1.34 ± 0.54^c^	1.44 ± 0.37^c^	2.39 ± 0.35^a^^b^
T_k_ (Nm.kg^−1^)	1.46 ± 0.83^c^	1.50 ± 0.97	1.87 ± 1.32^a^
T_h_ (Nm.kg^−1^)	4.97 ± 2.01^b^^c^	5.54 ± 2.16^a^^c^	6.34 ± 2.91^a^^b^

Values are mean ± sd.

a Significantly different from TS.

b Significantly different from VS.

c Significantly different from JS.

Peak flexion (θ) and velocity (ω) during the eccentric (Ecc) and concentric (Con) phases and peak torque (T) at the ankle (a), knee (k) and hip (h) are shown.

Peak plantar flexor moment was significantly greater in JS than TS and VS (*p* = 0.000 and 0.005, respectively) but was not different between TS and VS (*p* = 0.515). Peak knee extensor moment was significantly greater in JS than TS (*p* = 0.037) but, again, no difference was observed between TS and VS (*p* = 0.162). Finally, peak hip extensor moment was significantly greater in JS than TS (*p* = 0.010) and significantly greater in VS than TS (*p* = 0.036).

MTU behavior, movement kinetics and muscle activity varied between the three conditions (Table 2). With all subjects and with all squatting speeds the tendon went through significant lengthening as it was loaded, and peak tendon strain occurred in the late concentric phase prior to unloading. During JS, which was performed at the fastest movement speed, peak tendon strain was significantly greater than during TS, the slowest movement speed (*p* = 0.041). However, no significant difference was found between VS and TS (*p* = 0.354; **Figure 3**). The greater tendon lengthening in JS occurred simultaneously with a greater F_T_ and RFD_T_ when compared to TS (*p* = 0.037 and 0.004, respectively), while no significant differences were observed between TS and VS (*p* = 1.000 and 0.717, respectively; **Figure 3**). VL muscle activity (sEMG_VL_) was also significantly greater in JS than TS (*p* = 0.048), however there was no significant difference between TS and VS (*p* = 0.780).

**Table 2:** **Tendon dynamics during slow-tempo (TS), volitional speed (VS), and jump (JS) squats performed with 60% of 1RM (***n*** = 10)**.

	**TS**	**VS**	**JS**
L_T_ (cm)	27.5 ± 2.7^b^	26.7 ± 3.2	28.2 ± 2.9^a^
F_T_ (kN)	2.35 ± 1.14^b^	2.34 ± 1.11	2.83 ± 1.13^a^
RFD_T_ (kNs^−1^)	5.54 ± 3.04^b^	6.61 ± 2.80	9.50 ± 4.27^a^

Values are mean ± sd.

a Significantly different from TS.

b Significantly different from JS.

**Figure 2:**
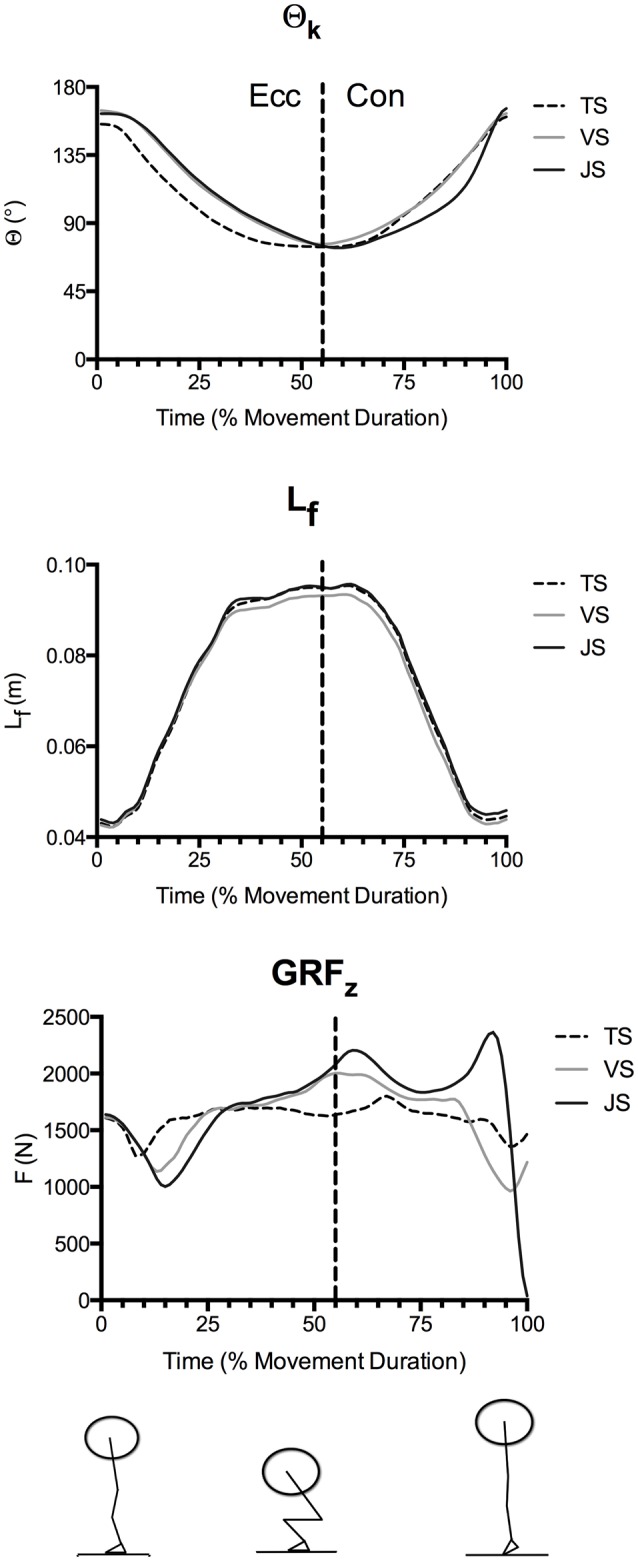
**Mean knee angle (Θ_**k**_), fascicle length (L_**f**_), and vertical ground reaction forces (GRF_**z**_) during parallel-depth slow-tempo (TS), volitional speed (VS), and jump (JS) squat conditions (***n*** = 10)**. Peak knee flexion occurred at 53.7 ± 6.1, 55.9 ± 4.9, and 59.3 ± 4.6% movement duration for TS, VS, and JS, respectively. Range of SEM at 20, 40, 60, 80, and 100% movement duration for Θ_k_ (0.12°−0.33°), L_f_ (0.46–1.87 cm) and GRF_z_ (140–487 N) are reported.

**Figure 3:**
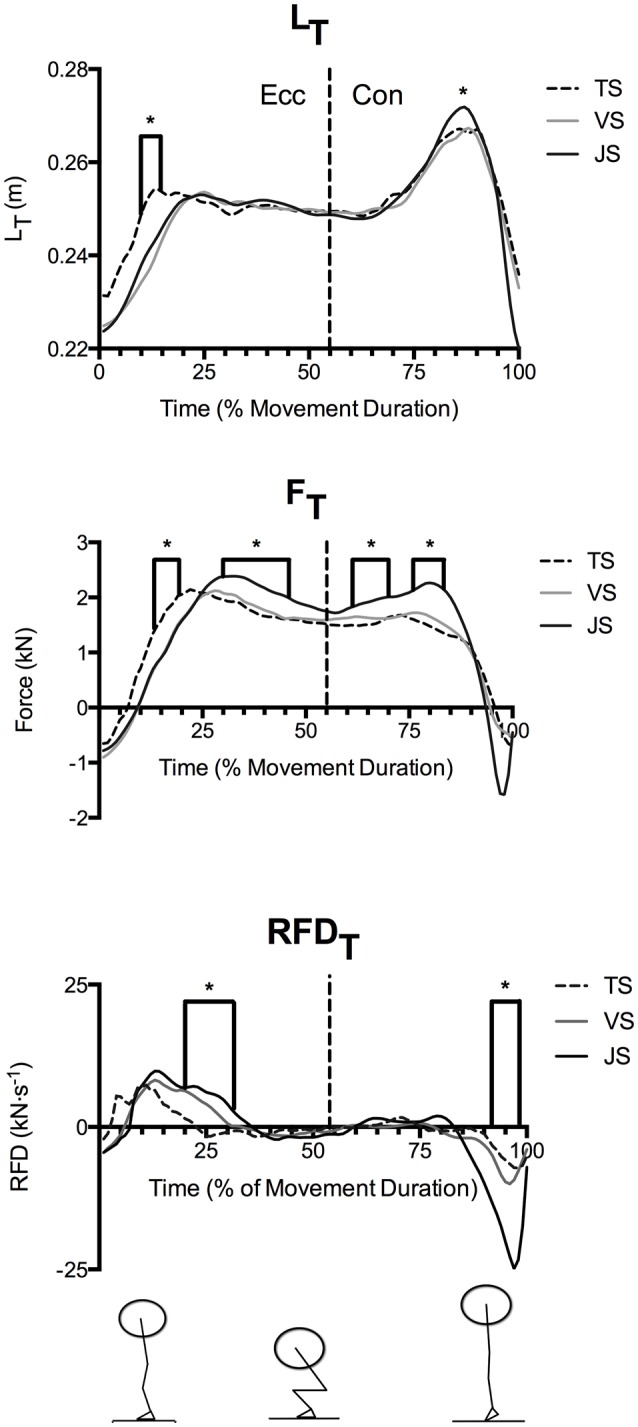
**Mean quadriceps tendon length (L_**T**_) and patellar tendon force (F_**T**_) and rate of force development (RFD_**T**_) during parallel-depth slow-tempo (TS), volitional speed (VS), and jump (JS) squat conditions (***n*** = 10)**. Range of SEM at 20, 40, 60, 80, and 100% movement duration for L_T_ (2.6–3.5 cm), F_T_ (0.46–1.87 kN) and RFD_T_ (0.70–6.30 kN·s^−1^) are reported. ^*^Indicates significant difference between TS and JS.

**Figure 4:**
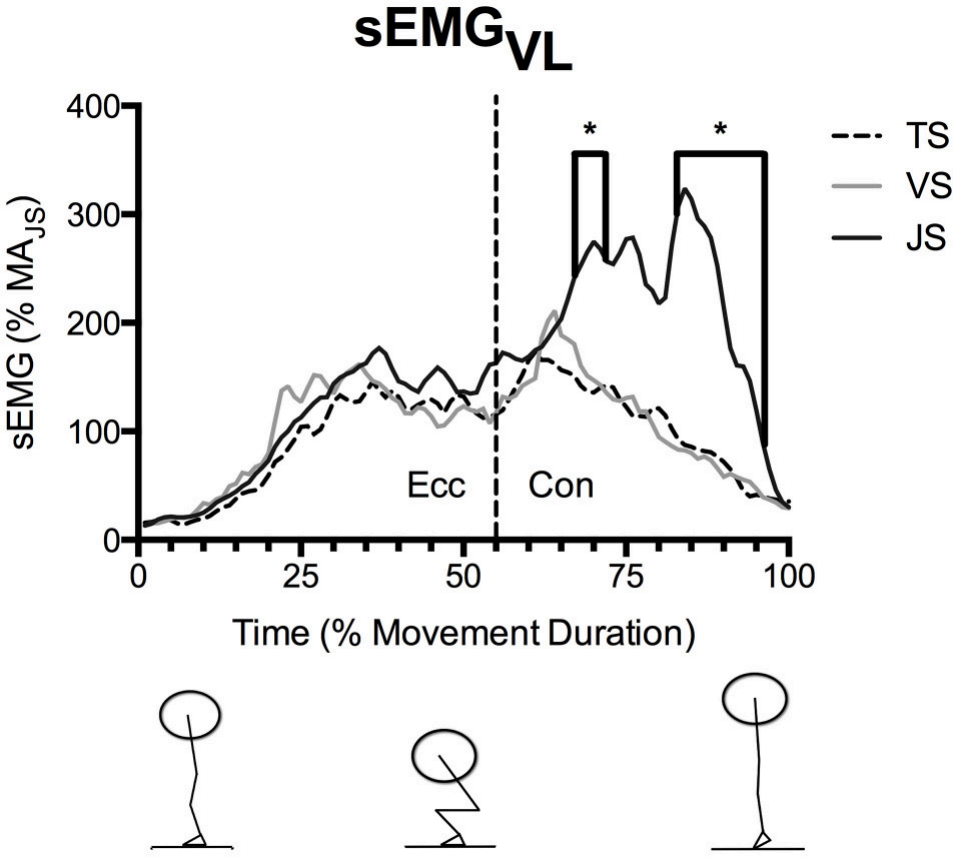
**Mean vastus lateralis EMG (sEMG_**VL**_) during parallel-depth slow-tempo (TS), volitional speed (VS), and jump (JS) squat conditions (***n*** = 10)**. Values are presented as a percentage of muscle activity during an unloaded jump squat. ^*^Indicates significant difference between TS and JS. Range of SEM at 20, 40, 60, 80, and 100% movement duration is 11.7–84.8% activity of the unloaded jump squat.

Time-normalized knee angle (Θ_k_), fascicle length, and vertical ground reaction force curves in TS, VS, and JS conditions are shown in Figure 2, time-normalized L_T_, F_T_, and RFD_T_ curves are shown in Figure 3 while time-normalized sEMG_VL_ curves are shown in Figure 4. Analysis of the L_T_ curve revealed that the tendon went through initial lengthening in the early eccentric phase at all movement speeds, with relatively little further change in until the early concentric phase when the tendon lengthened rapidly before shortening late in the concentric phase (Figure 3). Comparisons of tendon lengthening throughout the movements revealed that the tendon lengthened significantly more during the early eccentric phase in TS than JS (10–14% movement duration; *p* < 0.05). Despite this, peak tendon lengthening was significantly greater in JS than TS (*p* = 0.041), occurred in the concentric phase (~75–90% movement time) in all subjects, and was followed by rapid shortening at the end of the movement (~90–100% movement time).

F_T_ demonstrated a similar temporal pattern as L_T_ (Figure 3), where F_T_ was significantly greater in TS than JS in the early eccentric phase (13–19% movement duration). However, F_T_ was significantly greater in JS than TS during the late eccentric phase (30–46% movement duration) and the early concentric phase (61–70 and 76–83% movement duration).

Nonetheless, RFD_T_ displayed a dissimilar temporal pattern in that RFD_T_ was greatest in the early eccentric phase. Following this, additional tendon loading occurred at low relative rates until rapid unloading occurred near the end of the eccentric phase (Figure 3). Furthermore, between-condition comparisons revealed that RFD_T_ was significantly greater in JS than TS in the early eccentric phase (20–31% movement duration) where peak values were achieved, however it was significantly greater in TS than JS in the late concentric phase (92–98% movement duration) at which point the tendon was rapidly unloaded.

sEMG_VL_ did not differ significantly between conditions during the eccentric phase (Figure 4), however sEGM_VL_ was significantly greater in JS than TS for the majority of the concentric phase (54–55, 67–71, and 83–96% of movement duration).

## Discussion

Tendon stress and strain during exercise may be important stimulants for long-term training adaptation as well as influencing movement performance (Arampatzis et al., 2007, 2010). The present study is the first to directly investigate how movement speed influences tendon stress and strain during complex, multi-joint exercise, using the commonly performed squat lift. The major finding of the present study was that manipulating speed during a loaded squat exercise significantly influenced tendon strain and force during the movement. Specifically, greater tendon lengthening (L_T_), forces (F_T_), and rates of force development (RFD_T_) were observed when the subjects were instructed to move with the intention to reach maximal concentric speed (Jump Squat condition: JS) than when instructed to move at a fixed, slow tempo (Tempo Squat condition: TS). Furthermore, time-normalized L_T_ and F_T_ curves displayed similar patterns, where an increase in load resulted in greater tendon lengthening. In contrast, RFD_T_ only differed between movement speeds during the early eccentric phase, thus did not significantly influence the peak tendon strain that occurred in the concentric phase. For these reasons it can be concluded that the elastic, rather than viscous, properties controlled the peak tendon strain experienced during the motion.

As movement durations differed between the jump and tempo squats, time-normalized curves were used to compare temporal differences in the dependent variables throughout the movement. Analysis of these curves revealed that the tendon was rapidly loaded and lengthened during the early eccentric phase (~0–33% of movement duration). During this phase of the exercise peak RFD_T_ was obtained and F_T_ and L_T_ both rapidly increased (see Figures 2–4). Subsequently, RFD_T_ decreased to a fraction of its previous value during the late eccentric phase and early concentric phase (~33–66% of movement duration), which resulted in a sustained high level of tendon force and a sustained tendon strain. During this time period, greater stiffness was observed in JS than VS because the tendon underwent similar strain to VS but experienced greater F_T_; this phenomenon in JS is consistent with a predominance of the tendon's viscous (i.e., rate dependent) properties. Finally, during the mid- and late-portions of the concentric phases the tendon underwent a second lengthening phase during which peak L_T_ was obtained. However, this second lengthening phase was characterized by relatively slow RFD_T_ and minimal changes in F_T_ compared to the eccentric phase. The tendon was subsequently rapidly unloaded and shortened (i.e., recoiled) back to its original length. These results clearly demonstrate that manipulation of movement speed with a fixed load can result in significant changes in tendon dynamics, which potentially influence long-term tendon adaptation in addition to its acute effect on movement power and efficiency.

While the time-normalized temporal patterns in L_T_, F_T_, and RFD_T_ were similar in JS and TS, comparison between conditions revealed that altering the speed of movement did change their magnitude. For instance RFD_T_ was significantly greater in JS than TS only during initial tendon loading (20–31% movement duration), which resulted in significantly greater F_T_ through most of the late eccentric phase (30–46% movement duration) in JS. However, despite the tendon experiencing greater F_T_ in JS, L_T_ did not differ between the two conditions during this movement phase, as would be expected of a purely elastic material. Interpretation of these results suggests that the tendon acted more as a viscoelastic structure than an elastic structure during the eccentric phase of the motion. Specifically, increasing squat movement speed reduced the magnitude of tendon strain during the eccentric phase despite a greater F_T_ and potentially resulting from the greater RFD_T_, thus demonstrating tendon viscous resistance. These results are similar to those previously observed during externally loaded ballistic knee extensions (Earp et al., 2014).

**Figure 5:**
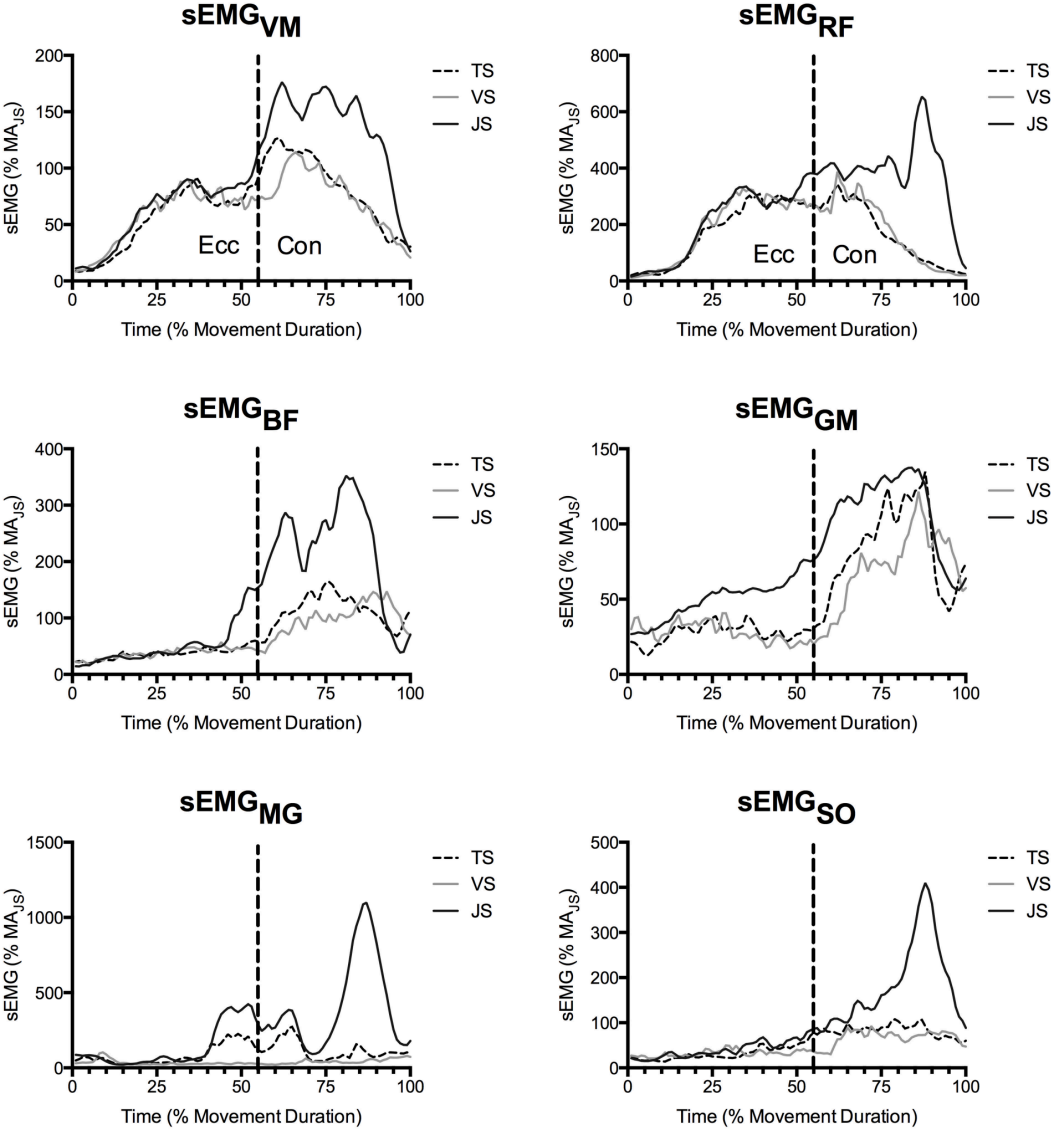
**Mean muscle activity of the vastus medialis (VM), rectus femoris (RF), biceps femoris (BF), gluteus maximus (GM), medial gastrocnemius (MG), and soleus (SO) as recorded by surface EMG (***n*** = 10)**. Values are presented as a percentage of muscle activity during an unloaded jump squat.

During the concentric phase, greater peak tendon force was observed in JS than TS (61–70 and 76–83% movement duration), which likely resulted from the goal of maximally accelerating the load into the flight phase in the JS condition (see ground reaction force traces in Figure 3). Nonetheless, RFD_T_ did not differ between conditions and was in fact only a fraction of the value observed at the end of the eccentric phase, despite there being a greater muscle activity (as measured using EMG; Figures 4, 5). Furthermore, the tendon underwent a second lengthening event during which peak tendon strain occurred, and which was significantly greater in JS than TS. Thus, the tendon's elastic properties apparently predominated in the concentric phase as the JS movement elicited a greater F_T_ and also a greater L_T_. The differing stress-strain behavior of the tendon between the concentric and eccentric phases might be explained by an alteration of tendon composition between these phases (Elliott et al., 2003). During initial eccentric loading intrinsic fluid will be forcefully pushed out of the collagen matrix resulting in significant viscous resistance. However, since force is sustained prior the second force event in the concentric phase viscous resistance would be greatly dampened because this fluid has already been lost during the initial lengthening without a chance to return. The result is that the tendon behaves as a more elastic structure during the subsequent concentric phase.

When comparing movement conditions it is important to put into context that the external load utilized in the present study (60% of 1RM load) is a training load that is normally utilized to elicit adaptations in muscular endurance in movements such as TS and VS in relatively healthy individuals or as part of an intermediate or late phase tendon rehabilitation after injury (Baechle and Earle, 2008). However, in movements such as JS, lighter external loads (e.g., 0–30% of 1RM load) are more common as the majority of studies have found that peak external power occurs at these loads in most individuals. Furthermore, while external load was controlled in this study, it is likely that in the TS movement, increasing external load would result in increases in both tendon strain (L_T_) and tendon load (F_T_). However, as previous works have found that a large external load moved as in a ballistic stretch shortening cycle exercise can elicit dynamic stiffening of the tendon, more research is needed to determine the influence of external loading on JS and VS movements.

Collectively, the results of the present study demonstrate that the tendon's viscous properties only significantly impacted MTU behavior during the initial eccentric loading phase during the large range of motion, loaded squat exercises. Because a significant F_T_ is maintained after this initial rapid loading, RFD_T_ during the concentric phase did not appear to influence tendon lengthening. Thus, the tendon behaved like a predominately elastic structure during the concentric phase when additional force was produced and muscle activity was sustained. These results support the null hypothesis that performing a loaded squatting exercise with slower movement speeds will not result in greater tendon lengthening. That is, the current results support previous observations during running and jumping tasks where the additional force required to accelerate the body in faster movements resulted in greater tendon lengthening, as would be predicted of an elastic structure (Finni et al., 2001; Ishikawa et al., 2007; Lichtwark et al., 2013; Malliaras et al., 2013).

When interpreting F_T_ and RFD_T_ temporal curves one important point to note is that negative (compressive) tendon forces were observed during movement initiation (~0–10% movement duration) and termination (~90–100% movement duration) when quadriceps femoris activity was minimal and antagonists may have activated in order to initiate the movement or decelerate the joint at the end of rapid extension to prevent knee hyperextension. However, the compressive forces reported would likely more closely represent an unloaded tendon than actual compressive force because equations used to calculate F_T_ do not take into account antagonist contribution, which may have contributed to joint toque during this time.

A secondary finding of the present study was that performing the squat lift at a self-selected speed without a pause (volitional speed; VS) did not result in a significant change in peak tendon strain compared to the performance of the squat with a deliberate pause at the lowest eccentric position (TS). These results are interesting in view of the greater ground reaction forces, joint velocities, joint accelerations and shorter movement durations observed in TS compared to VS. As these differences did not translate to significant differences between TS and VS in F_T_ (2.345 vs. 2.340 kN; *p* = 1.000) or RFD_T_ (5.53 vs. 6.61 kN•s^−1^, *p* = 0.717), we are unable to directly address our secondary hypothesis that moving at a deliberate slow-fixed speed would result in greater tendon strain than moving at volitional speed (27.5 vs. 28.2 cm; *p* = 0.354). However, it can be concluded that performing the loaded squat exercise at a predetermined slow tempo using a pause with a 2-s eccentric phase, 1-s pause, and 2-s concentric phase did not affect peak tendon stress or strain compared to a squat in which no instructions as to movement speed were given. It should be noted that while no effect of prescribing a longer movement duration was observed in the present study it is possible that longer movement durations that significantly reduce RFD_T_ might potentially result in a greater tendon strain, and this should be explicitly examined in future studies. Furthermore, longer movement durations will increase the tissue's time under tension, which may speculatively influence tendon adaptation. However, as the decreasing movement speed will reduce the total number of repetitions that can be performed prior to volitional fatigue, decreasing movement speed beyond the constraints of the present study may decrease F_T_. For these reasons, further research is necessary to explore this relationship in detail.

Quantifying the stresses and strains experienced by tendons during commonly-performed exercises such as the squat lift allows the formulation of hypotheses regarding potential long-term adaptations in tendon mechanical properties, since peak tendon strain has been directly linked to adaptations in tendon stiffness (Arampatzis et al., 2007, 2010). This has direct relevance in the clinical setting, such as in the treatment of tendinopathy or the prevention of acute tendon injury; exercises that elicit maximal tendon strain might evoke the greatest improvements in tendon stiffness and tensile strength as well as reductions in pain and injury risk. Indeed, squat lift training is the most common non-surgical treatment for patellar tendinopathy (Malliaras et al., 2013; Joseph and Denegar, 2015). While previous studies have demonstrated that tendon strain increases with movement speed in running and (relatively small range of motion) jumping exercises (Finni et al., 2001; Ishikawa et al., 2007; Lichtwark et al., 2013; Malliaras et al., 2013), the present study is the first to observe such a relationship in a commonly-performed exercise with a fixed external load.

In support of the finding of the present study that the influence of FT and RFDT on LT differs throughout the movement, supplemental correlation analysis for segmented time windows throughout the movement for TS (Table 3) and JS (Table 4) were performed. However, these *post-hoc* tests lacked necessary statistical power to provide significance, but as they do provide some additional information as to the strength and directional nature of possible relationships and temporal locations where significance may occur if future research is performed with adequate statistical power (*p* = 0.07 and 0.08) this information has been included (Tables 3, 4).

**Table 3:** **Relationship between quadriceps tendon length (L_**T**_) and patellar tendon force magnitude (F_**T**_) and rate (RFD_**T**_) during a loaded fixed tempo squat (TS)**.

**L_T_ —TS**										
	**0–10%**	**10–20%**	**20–30%**	**30–40%**	**40–50%**	**50–60%**	**60–70%**	**70–80%**	**80–90%**	**90–100%**
F_T_	*r* = 0.250	*r* = 0.349	*r* = 0.477	*r* = 0.375	*r* = 0.374	*r* = 0.278	*r* = 0.204	*r* = 0.190	*r* = 0.112	*r* = 0.224
	*p* = 0.49	*p* = 0.32	*p* = 0.16	*p* = 0.29	*p* = 0.29	*p* = 0.44	*p* = 0.57	*p* = 0.60	*p* = 0.76	*p* = 0.53
RFD_T_	*r* = 0.556	*r* = 0.326	*r* = −0.184	*r* = −0.710	*r* = −0.128	*r* = −0.580	*r* = −0.352	*r* = 0.146	*r* = −0.103	*r* = −0.549
	*p* = 0.10	*p* = 0.36	*p* = 0.61	*p* = 0.85	*p* = 0.72	*p* = 0.08	*p* = 0.32	*p* = 0.69	*p* = 0.78	*p* = 0.10

Values are reported for 10% duration intervals throughout the movement.

**Table 4:** **Relationship between quadriceps tendon length (L_**T**_) and patellar tendon force magnitude (F_**T**_) and rate (RFD_**T**_) during a loaded jump squat (JS)**.

**L_T_ —JS**										
	**0–10%**	**10–20%**	**20–30%**	**30–40%**	**40–50%**	**50–60%**	**60–70%**	**70–80%**	**80–90%**	**90–100%**
F_T_	*r* = 0.505	*r* = 0.568	*r* = 0.558	*r* = 0.495	*r* = 0.495	*r* = 0.497	*r* = 0.360	*r* = 0.245	*r* = 0.307	*r* = 0.227
	*p* = 0.13	*p* = 0.09	*p* = 0.09	*p* = 0.17	*p* = 0.15	*p* = 0.14	*p* = 0.31	*p* = 0.50	*p* = 0.39	*p* = 0.53
RFD_T_	*r* = 0.596	*r* = −0.320	*r* = −0.097	*r* = −0.165	*r* = 0.079	*r* = −0.153	*r* = −0.137	*r* = −0.270	*r* = 0.174	*r* = −0.192
	*p* = 0.07	*p* = 0.37	*p* = 0.65	*p* = 0.65	*p* = 0.83	*p* = 0.67	*p* = 0.71	*p* = 0.45	*p* = 0.63	*p* = 0.60

Values are reported for 10% duration intervals throughout the movement.

It should be noted that the methods adopted in the present study have several important limitations that should be considered when assessing its results. First, VL muscle-tendon unit length, patellar tendon moment arm length, and segmental radii of gyration locations that were used to calculate quadriceps tendon length, and patellar tendon force and rate of force development were taken from previously published cadaver based equations and not directly measured in the present study (Dempster, 1955; Visser et al., 1990; Winter, 1990; Robertson et al., 2004). While we adopted a within-subject design where systematic errors based on possibly inaccurate assumptions would not affect comparisons between movements it is possible that the calculated values for these variables will be different than the true mechanical values. Another limitation was that between ~20 and 80% movement duration fascicle length was partially extrapolated using a validated and reliable method (Kurokawa et al., 2001; Abellaneda et al., 2009; Earp et al., 2014). However, it is possible that some inaccuracies may have occurred when calculating L_T_ during this portion of the motion. Finally, in order to accurately and reliability measure changes in L_T_ in hypertrophied individuals during high intensity and ballistic movements it was necessary to utilize anchored ultrasound images of the muscle belly. However, as previously stated calculation of L_T_ from this method can result in some inaccuracies because of the assumption that tissues both proximal and distal to the fascicle being measured are considered tendinous tissue in series (Abellaneda et al., 2009). However, it is well-established that the stress-strain response of tissue will vary along the length of the tendon and muscle, which may influence our results.

In summary, the present results indicate that performing a loaded squat at a faster movement speed results in a greater quadriceps tendon force (i.e., stress) and lengthening (i.e., strain) than when it is performed at a slow tempo. Furthermore, because RFD_T_ was several-fold greater in the early eccentric phase and peak tendon lengthening occurred in the concentric phase, the anticipated minimization of tendon lengthening that may result from the tendon's viscous properties was not clearly observed, and thus the tendon appeared to act as a predominately elastic structure during the concentric phase. As large range of motion resistance training exercises have been established as potent stimuli for tendon adaptation, manipulation of acute program variables such as movement speed may allow for stimulus optimization for tendon adaptation, particularly in those suffering from tendinopathy or who require increased tendon stiffness for improved movement performance. A next step in the research process is to determine whether the greater tendon lengthening evoked by jump squat exercise results in greater chronic adaptations in the tendon when compared to slow-tempo (i.e., traditional) squat lift training.

## Author contributions

JE acted as the primary investigator for this study and wrote the manuscript. JE, RN, PC, and AB were instrumental in study design, data collection and analysis, interpretation of results, and reviewing the manuscript.

### Conflict of interest statement

The authors declare that the research was conducted in the absence of any commercial or financial relationships that could be construed as a potential conflict of interest.

## References

[B1] Abellaneda S. Guissard N. Duchateau J. (2009). The relative lengthening of the myotendinous structures in the medial gastrocnemius during passive stretching differs among individuals. J. Appl. Physiol. 106, 169–177. 10.1152/japplphysiol.90577.2008 18988765

[B2] Arampatzis A. Karamanidis K. Albracht K. (2007). Adaptational responses of the human Achilles tendon by modulation of the applied cyclic strain magnitude. J. Exp. Biol. 210, 2743–2753. 10.1242/jeb.003814 17644689

[B3] Arampatzis A. Peper A. Bierbaum S. Albracht K. (2010). Plasticity of human Achilles tendon mechanical and morphological properties in response to cyclic strain. J. Biomech. 43, 3073–3079. 10.1016/j.jbiomech.2010.08.014 20863501

[B4] Baechle T. R. Earle R. W. (2008). Essentials of Strength Training and Conditioning, 3rd Edn. Champaign, IL: Human Kinetics.

[B5] Bobbert M. F. (2001). Dependence of human squat jump performance on the series elastic compliance of the triceps surae: a simulation study. J. Exp. Biol. 204, 533–543. 11171304 10.1242/jeb.204.3.533

[B6] Bohm S. Mersmann F. Tettke M. Kraft M. Arampatzis A. (2014). Human Achilles tendon plasticity in response to cyclic strain: effect of rate and duration. J. Exp. Biol. 217, 4010–4017. 10.1242/jeb.112268 25267851

[B7] Cormie P. McBride J. M. McCaulley G. O. (2008). Power-time, force-time, and velocity-time curve analysis during the jump squat: impact of load. J. Appl. Biomech. 24, 112–120. 10.1123/jab.24.2.112 18579903

[B8] Dempster W. (1955). Space Requirements of the Seated Operator: Geometrical Kinematic, and Mechanical Aspects of the Body with Special Reference to the Limbs. Springfield, OH: Wright Air Development Center Technical Report.

[B9] Earp J. E. Newton R. U. Cormie P. Blazevich A. J. (2014). The influence of loading intensity on muscle-tendon unit behavior during maximal knee extensor stretch shortening cycle exercise. Eur. J. Appl. Physiol. 114, 59–69. 10.1007/s00421-013-2744-2 24150780

[B10] Elliott D. M. Robinson P. S. Gimbel J. A. Sarver J. J. Abboud J. A. Iozzo R. V.. (2003). Effect of altered matrix proteins on quasilinear viscoelastic properties in transgenic mouse tail tendons. Ann. Biomed. Eng. 31, 599–605. 10.1114/1.1567282 12757203

[B11] Ettema G. J. (1996). Mechanical efficiency and efficiency of storage and release of series elastic energy in skeletal muscle during stretch-shorten cycles. J. Exp. Biol. 199, 1983–1997. 8831144 10.1242/jeb.199.9.1983

[B12] Ettema G. J. (2001). Muscle efficiency: the controversial role of elasticity and mechanical energy conversion in stretch-shortening cycles. Eur. J. Appl. Physiol. 85, 457–465. 10.1007/s004210100464 11606015

[B13] Finni T. Ikegawa S. Lepola V. Komi P. (2001). *In vivo* behavior of vastus lateralis mucle during dynamic performance. Eur. J. Sport Sci. 85, 1–13. 10.1080/17461390100071101

[B14] Finni T. Ikegawa S. Lepola V. Komi P. (2003). Comparison of force-velocity relationships of vastus lateralis muscle in isokinetic and in stretch-shortneing cycle exercises. Acta Physiol. Scand. 177, 483–491. 10.1046/j.1365-201X.2003.01069.x 12648166

[B15] Gosset J. F. Piscione J. Lambertz D. Perot C. (2009). Paired changes in electromechanical delay and musculo-tendinous stiffness after endurance or plyometric training. Eur. J. Appl. Physiol. 150, 131–139. 10.1007/s00421-008-0882-8 18853177

[B16] Hansen P. Aagaard P. Kjaer M. Larsson B. Magnusson S. P. (2003). Effect of habitual running on human Achilles tendon load-deformation properties and cross-sectional area. J. Appl. Physiol. 95, 2375–2380. 10.1152/japplphysiol.00503.2003 12937029

[B17] Hermie H. J. Freriks B. (1996). The State of the Art on Sensors and Sensor Placement Procedures for Surface Electromyography: A Proposal for Sensor Placement Procedures. Enschede: Roessingh R&D.

[B18] Ishikawa M. Finni T. Komi P. (2003). Behaviour of vastus lateralis muscle-tendon during high intensity SSC exercises *in vivo*. Acta Physiol. Scand. 178, 205–213. 10.1046/j.1365-201X.2003.01149.x 12823178

[B19] Ishikawa M. Pakaslahti J. Komi P. V. (2007). Medial gastrocnemius muscle behavior during human running and walking. Gait Posture 25, 380–384. 10.1016/j.gaitpost.2006.05.002 16784858

[B20] Joseph M. F. Denegar C. R. (2015). Treating tendinopathy perspective on anti-inflammatory intervention and therapeutic exercise. Clin. Sports Med. 34, 363–374. 10.1016/j.csm.2014.12.006 25818719

[B21] Kongsgaard M. Qvortrup K. Larsen J. Aagaard P. Doessing S. Hansen P.. (2010). Fibril morphology and tendon mechanical properties in patellar tendinopathy: effects of heavy slow resistance training. Am. J. Sports Med. 38, 749–756. 10.1177/0363546509350915 20154324

[B22] Kubo K. Kanehisa H. Fukunaga T. (2001). Effect of different duration isometric contraction on tendon elasticity in human quadriceps muscles. J. Physiol. 536, 649–655. 10.1111/j.1469-7793.2001.0649c.xd 11600697 PMC2278867

[B23] Kurokawa S. Fukunaga T. Fukashiro S. (2001). Behavior of fascicles and tendinous structures of human gastrocnemius during vertical jumping. J. Appl. Physiol. 90, 1349–1358. 11247934 10.1152/jappl.2001.90.4.1349

[B24] Lichtwark G. A. Cresswell A. G. Newsham-West R. J. (2013). Effect of running on human Achilles tendon length-tension properties in the free and gastrocnemius components. J. Exp. Biol. 216, 4388–4394. 10.1242/jeb.094219 24031068

[B25] Lichtwark G. A. Wilson A. M. (2007). Is Achilles tendon compliance optimised for maximum muscle efficiency during locomotion? J. Biomech. 40, 1768–1775. 10.1016/j.jbiomech.2006.07.025 17101140

[B26] Malliaras P. Kamal B. Nowell A. Farley T. Dhamu H. Simposon V.. (2013). Patellar tendon adaptation in relation to load-intensity and contraction type. J. Biomech. 46, 1893–1899. 10.1016/j.jbiomech.2013.04.022 23773532

[B27] Robertson D. G. E. Caldwell G. E. Hamill J. Kamen G. Whittlesey S. N. (2004). Research Methods in Biomechanics. Champlain, IL: Human Kinetics.

[B28] Roeleveld K. Baratta R. V. Solomonow M. van Soest A. G. Huijing P. A. (1993). Role of tendon properties on the dynamic performance of different isometric muscles. J. Appl. Physio. 74, 1348–1355. 8482677 10.1152/jappl.1993.74.3.1348

[B29] Visser J. J. Hoogkamer J. E. Bobbert M. F. Huijing P. A. (1990). Length and moment arm of human leg muscles as a function of knee and hip-joint angles. Eur. J. Appl. Physiol. 61, 453–460. 10.1007/BF00236067 2079066

[B30] Wiesinger H. P. Kösters A. Müller E. Seynnes O. R. (2015). Effects of increased loading on *in vivo* tendon properties: a systematic review. Med. Sci. Sport Exerc. 47, 1885–1895. 10.1249/MSS.0000000000000603 25563908 PMC4535734

[B31] Winter D. A. (1990). Biomechanics & Motor Control of Human Movement, 4th Edn. New York, NY: John Wiley & Sons Inc.

